# Analysis of Continuous Steering Movement Using a Motor-Based Quantification System

**DOI:** 10.3390/s121216008

**Published:** 2012-11-22

**Authors:** Hsin-Min Lee, Ping-Chia Li, Shyi-Kuen Wu, Jia-Yuan You

**Affiliations:** 1 Department of Physical Therapy, I-Shou University, No.8, Yida Rd., Jiaosu Village, Yanchao District, Kaohsiung City 82445, Taiwan; E-Mail: hmlee@isu.edu.tw; 2 Department of Occupational Therapy, I-Shou University, No.8, Yida Rd., Jiaosu Village, Yanchao District, Kaohsiung City 82445, Taiwan; E-Mail: pingchia@mail2000.com.tw; 3 Department of Physical Therapy, HungKuang University, No. 1018, Sec. 6, Taiwan Boulevard, Shalu District, Taichung City 43302, Taiwan; E-Mail: skwu@sunrise.hk.edu.tw

**Keywords:** continuous steering movement, torque measurement, hand contact information, steering cycle

## Abstract

Continuous steering movement (CSM) of the upper extremity (UE) is an essential component of steering movement during vehicle driving. This study presents an integrated approach to examine the force exertion and movement pattern during CSM. We utilized a concept similar to the isokinetic dynamometer to measure the torque profiles during 180°/s constant-velocity CSM. During a steering cycle, the extremity movement can be divided into stance and swing phases based upon the hand contact information measured from the hand switch devices. Data from twelve normal young adults (six males and six females) showed that there are three typical profiles of force exertion. The two hands exhibit similar time expenditures but with asymmetric force exertions and contact times in both the clockwise (CW) and counterclockwise (CCW) steering cycles. Both hands contribute more force but with less contact time in their outward CSM directions (*i.e.*, CW for the right hand and CCW for the left hand). These findings help us to further understand CSM and have a number of important implications for future practice in clinical training. Considerably more research is required to determine the roles of the various shoulder muscles during CSM at various speeds.

## 1. Introduction

Vehicle driving is a common skill of daily living activity in many countries. In Taiwan, almost two-thirds of adults have driving licenses, and average car ownership reached 0.88 per household [1] in 2012. Car ownership rates higher than 500 per 1,000 people are found in more than thirty different countries [2]. Steering wheels control a vehicle’s trajectory, and operating steering wheels is a major component of the driving task. To make a hard turn onto a street or to make a U-turn to reverse direction, continuous steering movement (CSM) is essential. Compared to the subtle steering adjustments during straight lane driving, CSM involves movements of almost all upper limb joints in a larger functional range of hand use. To make the wheel rotate, steering torque is exerted on the wheel by the movement of proximal joints of the upper extremities (UEs) with the tangent force applied on the rim of the wheel via hand grasping. During CSM, the UE muscles contract to generate the necessary force or torque, and these muscle activities are a good candidate for UE exercises. Further, CSM features a coordinated and reciprocal use of both hands that is suitable for the bimanual task of rehabilitation training [3,4]. It is interesting and necessary to know the details of the force exertion and movement pattern during CSM to understand the feasibility of using CSM in a therapeutic program for clinical training of disabled patients.

Previous studies have used observation of hand positions [5], video-based motion analysis [6,7], and the electromyography (EMG) technique [8] to understand the movement pattern of the UEs during simulated driving. To see the force exertion during driving, the grip force [9,10] and static torque [11,12] as well as the dynamic [13] steering torque were analyzed. However, previous research was mainly concerned with the issues of driving comfort and ergonomics [11,12] during a small-range steering movement in simulated lane-driving; fewer studies have focused on the force pattern during a larger range of steering [13] or CSM. As the torque measurement is contaminated by the moment inertia of the wheel movement, it is difficult to measure the exact torque profile generated from the driver in dynamic movement or CSM. The measurement is also affected by the design of the torque sensor as the continuous rotation of the steering wheel causes the cords of the torque sensor to become twisted. Moreover, there has been little discussion about movement patterns including the hand contact pattern during the larger-range steering or CSM.

For clinical purposes, the steering exercise might be a good training program for patients suffering from the effects of conditions such as traumatic brain injury or a stroke [14–16]. Before we apply this treatment to pathological patients, the first priority is to understand the characteristics of the steering force exertion and movement pattern. Hence, the aims of the study are twofold. First, we try to develop an integrated measurement system to analyze the torque profile and movement pattern during continuous steering movement. A concept similar to the torque measurement during isokinetic movement [17] was implemented. The steering torque is measured during the motor-driven rotation of a steering wheel at a constant angular velocity to eliminate the inertia problem. Second, the torque profile and movement pattern (temporal and spatial parameters) during CSM are measured and analyzed for 12 normal adults to understand the characteristics of the steering movement pattern. We select the common steering maneuver (cross-handed) to elucidate the force exertion and movement pattern during both clockwise (CW) and counterclockwise (CCW) directions of steering. The differences in hand use between bilateral UEs are also compared and discussed.

## 2. Experimental Methods

### 2.1. System Setup

**Figure 1.**
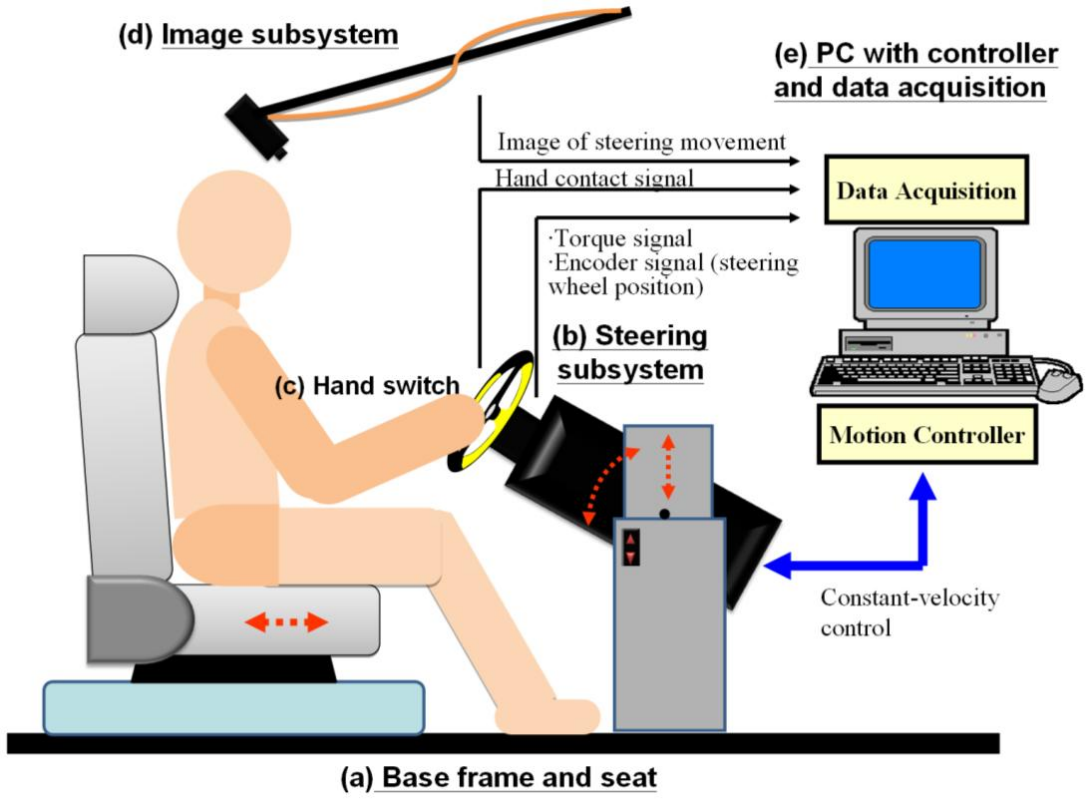
The integrated measurement system for characterizing steering movement. (**a**) Adjustable base frame and seat for subjects. (**b**) Steering subsystem for torque and position recording. (**c**) Hand switch devices for measuring the status of hand contact on the steering wheel. (**d**) Image subsystem that provides video of the steering movement. (**e**) Personal computer with controller board and data acquisition subsystem.

An integrated system with kinetic and kinematic measurements was developed to analyze the continuous steering movement (as shown in Figure 1). Details of the subparts are described as following.

#### 2.1.1. Adjustable Base Frame and Seat

As shown in Figure 1(a), the measuring subsystem with a steering wheel was set on a base frame that allows adjustment of the height and tilt angle of the interface. Along with the adjustable seat, the distance, height, and orientation of the steering wheel can be adjusted to fit different subjects and steering postures.

#### 2.1.2. Steering Subsystem

**Figure 2.**
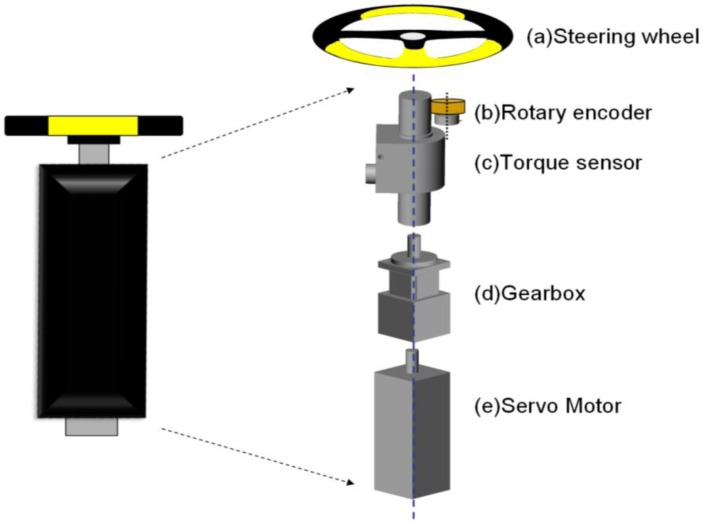
The steering subsystem. (**a**) Universal steering wheel. (**b**) Rotary encoder (OEW2-36-2MD, Nemicon, Inc.) with a resolution of 14,400 ppr for position recording of the steering wheel. (**c**) Rotary torque sensor with an integral slip ring assembly and a maximal measured range of 200 N·m. (**d**) Gearbox with a 100:1 ratio used to increase the torque while reducing the speed of the servo motor. (**e**) Servo motor with an encoder resolution of 10,000 ppr and a maximal rotation speed of 3,500 rpm.

The steering subsystem [Figure 1(b)] was designed to measure the position of the steering wheel and the steering torque during constant-velocity steering movement. Figure 2 shows a diagram of the steering subsystem. Via a rotary torque sensor (DR-2, Lorenz, Inc., Alfdorf, Germany), a 13-inch diameter steering wheel with a grip circumference of about 0.102 m was connected to a 100-to-1 gearbox and a DC servo motor (ASM-T04L250, Delta Electronics, Inc., Taipei, Taiwan). The torque sensor with an integral slip ring assembly can sense the coupled torsion torque between the wheel and the motor, even when the wheel continuously rotates through several full rotations during CSM. The servo motor controlled by the controller in the personal computer (PC) can precisely rotate the steering wheel at constant velocity at a maximum of 35 rpm (210°/s). Besides an internal encoder in the motor, a rotary encoder with a mounted rubber wheel on the shaft [the beige part in Figure 2(b)] was used to record the angular position of the steering wheel. If the rubber wheel and the rotary shaft of the torque sensor have identical circumferences, the encoder and the steering wheel can be made to rotate synchronously by joining them together in a rigid fashion. All components of the subsystem were placed and secured inside a rigid stainless steel box (the black component in Figure 2), which was fixed on the support frame.

#### 2.1.3. Hand Switch Devices

**Figure 3.**
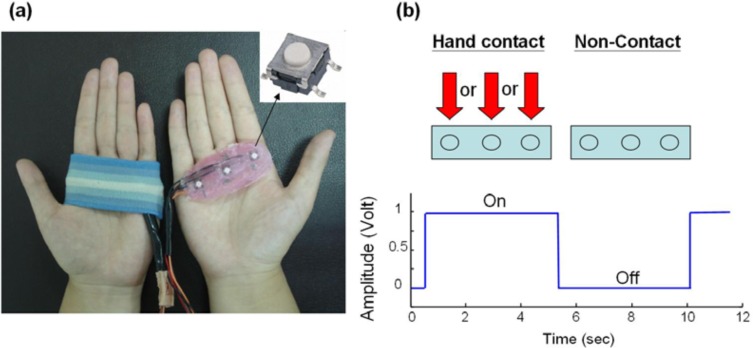
The hand switch devices. (**a**) Ultra-thin button switches were used to sense the contact status of both hands (where the wristband has been removed from the right hand in order to display the button switches). (**b**) When any of the button switches senses hand contact, this information is represented as an “on” signal.

To detect the exact time and duration that the subject’s hands were in contact with the steering wheel, hand switch devices were used (as shown in Figure 3). Three ultra-thin button switches (5 × 5 × 2.5 mm) encapsulated within two thin plastic membranes were put on each palm in the area of the metacarpal heads and wrapped with a soft wristband [Figure 3(a)]. The status of hand contact on the steering wheel can be encoded as an on-off signal (1 and 0) during steering movement, as shown in Figure 3(b). The hand contact signal also helps to isolate the repetition period of the steering cycle (to be discussed in detail in Section 2.3.1) from a sequence of sensor data.

#### 2.1.4. Image Subsystem

A monochrome camera (scA640-70fm, Basler, Inc., Frankfurt, Germany) was also used to record the steering movement [Figure 1(d)]. The camera captured images of the steering wheel area with a sampling rate of 10 frame/s and a resolution of 640 × 480 pixels and sent the resulting images to the PC through an IEEE 1394b card.

#### 2.1.5. PC-Based Controller and Data Acquisition Subsystem

**Figure 4.**
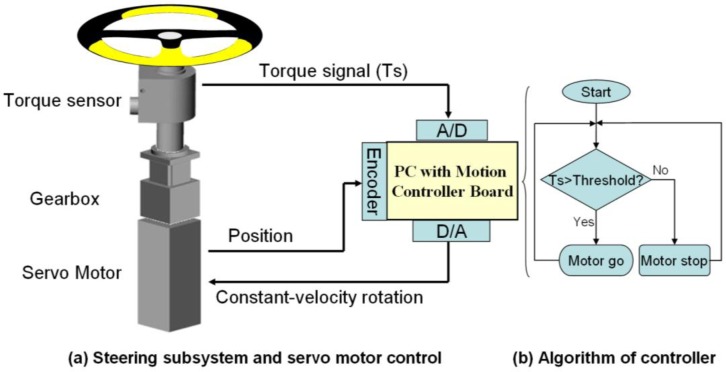
Control flow diagram for the continuous steering movement. (**a**) Steering subsystem provides the position and torque of the steering wheel to the controller and receives the command to rotate it at a constant velocity if the criterion in (**b**) is met. The criterion for rotation is that the steering torque must be larger than a preset value (0.5 N·m in this study).

As shown in Figure 1(e), a personal computer was used to administer the data acquisition and servo motor control. The measured torque, position, and hand switch signals were acquired by the PC through a 12-bit A/D converter (PCI-6259, National Instruments, Inc., Austin, TA, USA) at a sampling rate of 1 kHz for storage and display of the data. A motion control board (PCI-7350, NI Inc.) was installed in the PC as a proportional integral-derivative (PID) controller to control the movement of the servo motor and to rotate the steering wheel at a constant velocity via a servo drive (ASD-A0421LA, Delta Electronics, Inc., Taipei, Taiwan). To ensure that the continuing rotation of the steering wheel comes from the exertion of the subject, we set a criterion for controlling the wheel movement. Figure 4 depicts the diagram for motor control of the steering wheel at a constant velocity of rotation. In a control loop operating at 16 kHz (checked every 62.5 μs), the servo motor rotates at a selected constant velocity only when the applied torque (*T*_s_) exceeds the predetermined threshold of 0.5 N·m. The torque threshold was tested and determined before the experiment, and it was found to be easy for all subjects to rotate the steering wheel continuously.

#### 2.1.6. Software Interface

A customized LabVIEW-based program (Version 8.5, National Instruments, Inc., Austin, TA, USA) with an integrated user interface provides for the setting of parameters, display of data, and saving of position, torque, and image signals during the experiment. Furthermore, the subject can consult the PC screen in order to visualize the amplitude of the applied torque to help them keep the steering wheel rotating continuously.

### 2.2. Experimental Design

Twelve right-handed young subjects (six male, six female, with ages ranging from 20 to 25 y/o) agreed to volunteer for the following experiment. All of the subjects had no previous neurological disorders and had had no orthopedic problems in the six months prior to the test. Before the testing, each subject was instructed in the testing procedure and was allowed five minutes of steering practice to determine the proper body position for continuously steering by adjusting the base frame or seat. The anthropometric relations between the subject and our system can be understood from the following measured data. The shoulder width (SJ2SJ) was larger than the diameter of the steering wheel in almost all of the subjects (except s4 and s5, who are rather short). A general rule for selecting the seat position is to find the position such that when the subject reaches his or her hand toward the wheel, the wrist is about to touch the upper edge of the wheel; the subjects then handle the steering wheel in a posture of slight elbow flexion. This position can also be determined by the smaller distance from the shoulder joint to the steering center (SJ2SC, ranging from 46 to 56 cm), compared to the UE length (SJ2FT, ranging from 65 to 78 cm). The fact that the center of the steering wheel is lower than the shoulder joint was noted in all subjects, as the distances of SC2FL (steering center to floor) are smaller than those of SJ2FL (shoulder joint to floor). The selected tilt angle (TA) of the steering wheel ranged from 69° to 78°. Details of the participants’ characteristics, including height, weight, anthropometric measurements, and the self-selected tilt angle of the steering wheel, are summarized in Table 1.

As the steering wheel driven by the servo motor does not rotate when the subject stops turning it (Figure 4), all the subjects were asked to maintain continuous rotation of the steering wheel without interruption and were monitored to confirm this behavior. All subjects continuously rotated the steering wheel with the cross-handed maneuver in both clockwise and counterclockwise directions for at least one minute at the testing steering velocity of 30 rpm (180°/s). The velocity of 180°/s was selected, as it reaches the velocity of the authors’ real driving experience during fast turning. To eliminate the effect of trunk involvement, the subjects were instructed to not leave the seat back and were monitored by the instructor during the CSM trials.

### 2.3. Data Analysis

#### 2.3.1. Steering Cycle

**Figure 5.**
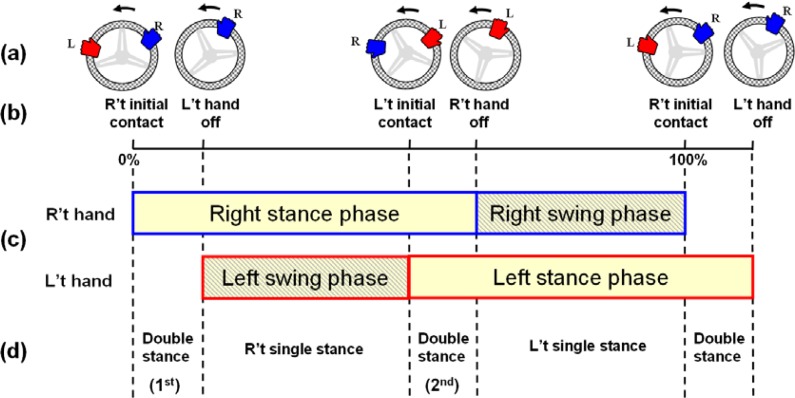

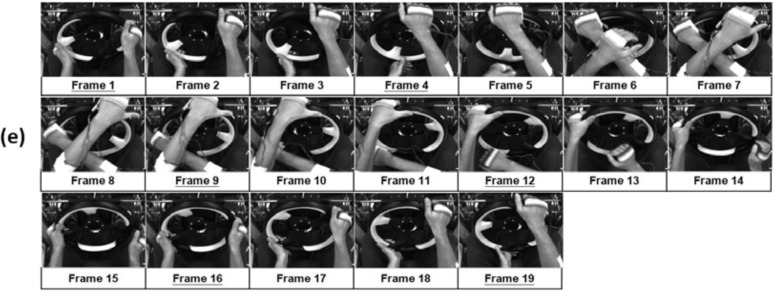
Steering cycle during a counterclockwise (CCW) steering movement. (**a**) Hand positions during the steering cycle. (**b**) Events in which the hands either made contact with the steering wheel or released the steering wheel. (**c**) Stance and swing phases for both hands. (**d**) Information with regard to the state of double or single contact. (**e**) Image series of a steering cycle with a rate of 10 frames/s. A sequence of 19 frames is shown to help us understand the steering movement during a 1.9-s period. The frame numbers that are underlined correspond to the transition events (initial contact and the hand-off) of the proposed steering cycle.

For each extremity, a steering cycle can be defined as the time period between two adjacent initial hand contacts, and it can be divided into contact and non-contact phases according to the hand switch signals. As shown in Figure 5, we provide an illustration of the steering cycle based on the information we have observed from the video captured by the image subsystem [Figure 5(e)]. During a steering cycle, the events and patterns of hand movements are similar to a gait cycle which includes a stance phase and a swing phase according to whether the hand/foot contacts the ground/wheel [18]. In a cycle of the CCW steering movement, the right hand initially contacts the wheel and holds and pulls it to move it to the left, while the left hand continues working on the wheel (the first double stance in Figure 5). In this case, as the left hand would, within a short duration, become ill-equipped to apply force, the hand would then unclench and swing across the right hand to a new position (left-swing phase), while the right hand would continue working on the wheel (beginning of the 2nd double stance). When the right hand also progresses to the point that it is in a poor position to apply force, the right-swing phase begins, and it ends at the next right initial contact.

#### 2.3.2. Analyzed Parameters

For each steering trial, at least eight successive steering cycles were selected for further analysis. By using the hand switch signals, all the data (torque, hand switch signals, and steering position) in each steering cycle was reduced to a normalized frame of 512 data points (100% steering cycle) for convenience. Further, the exerted torque signal and the displacement signal of the encoder were rectified to present the absolute values in the following analysis. We analyzed the continuous steering movement from the viewpoints of both the kinetic and kinematic aspects for each steering direction. In the kinetic aspect, we derived the averaged amplitude of the torque during the single stance phase for each hand to understand the contribution of the right and left hands to the torque profile. The averaged amplitude of the torque, *T*_amp_, was defined as:

(1) $${\text{T}}_{\text{amp}}=\frac{\sum_{n=1}^{k}T\left[n\right]}{k}$$

where *T*[*n*] represents the torque amplitude of the *n*th data point and *k* denotes the duration of the torque (in units of data sample points) during the single stance phase.

In the kinematic aspect, the temporal parameters include the cycle time, stance time, swing time, and stance ratio. Cycle time, stance time, and swing time are defined as the durations of a steering cycle, stance phase, and swing phase respectively. The stance ratio is the ratio of the stance time to the cycle time. For the spatial parameters, the averaged rotary displacements for each hand were analyzed in units of complete rotations. Furthermore, the video from the image system was also included and helped to observe and verify the sequence of movements of the upper limbs. In the statistical analysis, a paired *t*-test was used to test the difference between the parameters derived for the two hands or the two steering directions. *P* values below 0.05 were considered statistically significant.

### 2.4. Issues of System Performance and Measurement

To ensure the reliable and valid measurement of kinetic and kinematic parameters, several approaches were taken. First, the continuous rotation of the steering wheel and hand switch signals were monitored online during each trial. Any data involving interruptions in the rotation or bad hand switch contacts were rejected, and the subject was asked to repeat the trial. Second, raw torque signal for all trials were screened by the authors to confirm the signal stability before further analysis. Torque parameters for a trial were averaged over at least eight steering cycles to reduce the possibility of random interference affecting the signal. Third, the prepared procedures including the position adjustment and the pretrial practice also help to enhance the validity of the measurement. Furthermore, the captured video from the digital video recorder was screened to confirm there is no any unwanted trunk involvement during CSM trials.

**Figure 6.**
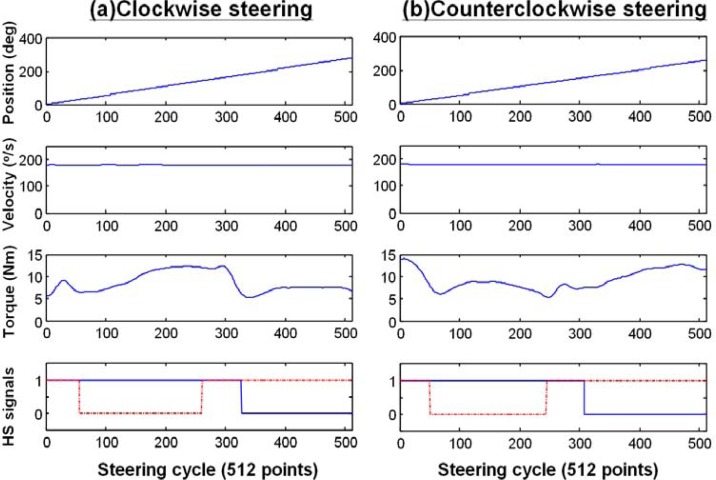
Processed data of position, velocity, torque, and hand switch (HS) signals from one subject (S7). The velocity and torque signals are processed with a low-pass filtering of 100 Hz. The duration and timing of all these data signals were extracted from the overall data set based upon the steering cycle obtained from the right HS signal and presented in the 512-point format. In the plots of the HS signals, the blue line and the red dash-dotted line represent the hand switch signals from the right and left hands, respectively. When both of the HS signals are “on” (corresponding to an HS signal value of 1), the system is in a double stance phase, which is followed by either a right single stance phase or a left single stance phase. (**a**) Clockwise steering. (**b**) Counterclockwise steering.

To demonstrate the system performance and measurement results, two typical examples of processed data based upon the steering cycles of the right hand were shown in Figure 6. During the CW and CCW steering trials, the displacements of the steering wheel were 282.23° and 258.00° with the derived averaged velocities of 180.56°/s and 180.42°/s. The linear displacement and constant velocity ensured the performance of the motor control. The steering torque during the cycle and the events of the first double stance (both hands on), right single stance, second double stance, and left single stance phases can be easily observed. Overall, the accurate measurement of signals (especially torque) during constant-velocity steering was confirmed.

## 3. Results

### 3.1. Kinetic Aspect: Torque Profile

**Figure 7.**
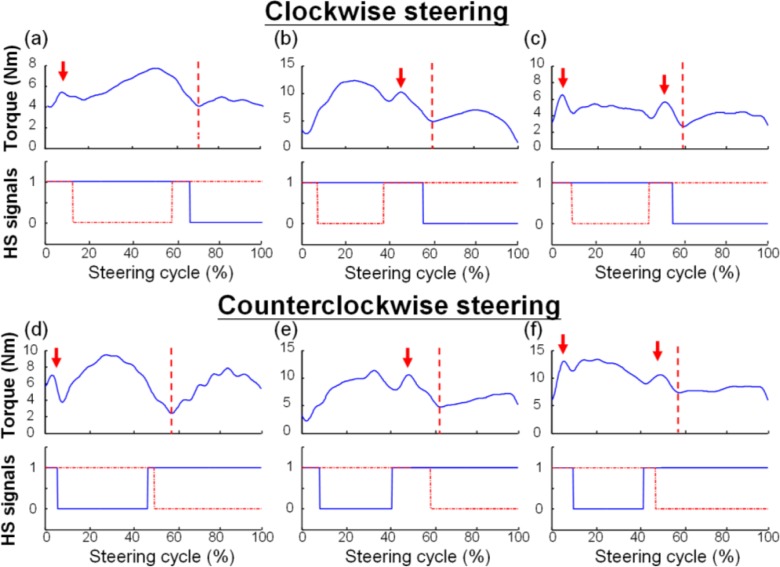
Three types of torque profiles (CW *vs.* CCW). Type 1: A ripple is only found in the first double stance of (**a**) and (**d**). Type 2: A ripple is only found in the second double stance of (**b**) and (**e**). Type 3: Ripples are found both in the first and second double stances of (**c**) and (**f**). In the HS signals, the blue line and red dash-dotted line represent the signals from the right and left hands, respectively.

As the steering wheel rotates at constant velocity, the steering torque can be measured without the effects of acceleration and deceleration of the wheel. As shown in Figure 7, we present three types of torque profiles to elucidate the features of force exertion in continuous steering movement. Compared to the torque profile of the CCW steering shown in Figure 6(b), we process the CCW data with respect to the left hand switch signal [Figure 7(d–f)]. Interestingly, the plots show that the torque signal can be divided into two major force exertions which are separated by a major dip after the second double stance (marked by the red vertical dash line) for both CW and CCW steering. In the torque profile before the major dip, a small ripple may appear in the first double stance [Figure 7(a,c,d,f)] or the second double stance [Figure 7(b,c,e,f)]. The small ripples are believed to be made by the hand contact of the swing hand. If the transition of the hand exchange is made more smoothly, the ripple effect might be diminished and may even disappear [second double stance of Figure 7(a,d) and the first double stance of Figure 7(b,e)].

To understand the contribution of hands to the torque profile, we compared the averaged amplitudes of the right and left hands in their single stance phase (Table 2). For CW steering, the torque amplitude is significantly larger for the right hand (8.23 ± 2.65 N·m), compared to the left hand (5.94 ± 1.51 N·m) with statistical significance (*P* < 0.05). In contrast, the amplitude of the torque in the left hand (8.04 ± 2.42 N·m) is significantly larger than that of the right hand (5.43 ± 1.42 N·m) for CCW steering (*P* < 0.05).

### 3.2. Kinematic Aspect

#### 3.2.1. Temporal Parameters

As summarized in Table 3, the averaged cycle times of the right and left hands are equivalent for either CW or CCW steering (about 1.60 and 1.53 s, respectively). However, a significantly longer stance time and a shorter swing time (*i.e.*, a higher stance ratio) were noted for the left hand for CW steering and in the right hand for CCW steering (all of which had *P* < 0.05).

#### 3.2.2. Spatial Parameters

In the study, the subject completed a steering cycle with less than one rotation (either right or left), varying from 0.53 to 0.99 rotations with an average of 0.8 ± 0.13 rotations in the CW direction and 0.76 ± 0.12 rotations in the CCW direction (where one complete rotation = 360°). There is no significant difference in cycle displacement between CW and CCW steering (*P* = 0.201).

## 4. Discussion

In this preliminary study, we developed a measurement system to analyze the torque profile and movement pattern of continuous steering movement. We utilized an idea similar to the basis of isokinetic dynamometry [17] to elucidate the force exertion pattern during a constant-velocity steering movement. Measuring the torque during a constant velocity motion of the steering wheel controlled by the motor has successfully eliminated the inertial problem of the wheel caused by the acceleration and deceleration of the rotating motor (as shown in Figures 6 and 7). The movement pattern of CSM, especially the hand contact on the steering wheel, was studied within a steering cycle (as shown in Figure 5). By using the hand switch devices, the temporal parameters including steering cycle time, stance time, swing time, and stance ratio could be analyzed. Our system has provided a unique approach to study the steering movement and has improved the understanding of UE movement during CSM.

The main results of the CSM analysis could be summarized in terms of the kinematic and kinetic aspects. In terms of the kinematics, CSM features the reciprocal movement of two upper extremities in an asymmetric movement pattern. The movement of each extremity can be partitioned into a stance phase, where the hand is in contact with the wheel, and a swing phase, where the hand is lifted and moved sideways to prepare another stance. In our study, each extremity for a given subject completes a cycle in a similar period of time but with an asymmetric ratio of hand contact (as indicated by the stance ratio listed in Table 3). A shorter duration of right hand contact in CW steering and left hand contact in CCW steering were found as the reachable areas of the steering wheel are different for the two hands in the CW and CCW directions. As a reciprocal movement pattern of the UEs, the pattern of time periods and events during a steering cycle are similar to those in the gait cycle of the lower extremities (LEs) [19], although CSM is predominately open-chain kinematic movements of the UEs [20], compared to the combination of open-chain and closed-chain movements of the LEs during a gait cycle. We hope the proposed steering cycle in the study helps researchers to better understand and study the continuous steering movement.

In terms of the kinetics, we found that all the subjects exhibited a similar torque profile pattern with two major exertions combined with one or two ripples in the transition of the hand change during a CSM cycle (Figure 7). These ripples, mainly occurring during the double stance phases, might indicate the proficiency of steering movement, which could be evaluated by the amplitude of the ripples. For a coordinated performance of the bimanual task, the exchange of hand contact might be smooth and the ripples might be small or disappear. Moreover, the contribution of torque exertion is asymmetric with right domination in the CW direction and left domination in the CCW direction (both are in their abduction directions). Interestingly, the hand that exerts more force on the rotation of the wheel has a shorter contact duration, while the other hand has a longer contact duration but a smaller contribution to the rotating torque (as indicated by the data in Tables 2 and 3). Independent of dominant hand use, the asymmetric contribution of the UEs indicates the differences of muscle use and functional range between the two hands. Although previous studies have found that the shoulder adductor is stronger than the abductor during isokinetic contractions [21,22], the combination of the effects of the constrained areas that are reachable by the two hands and the effective muscle length on maximal exertion largely contributed to the asymmetric pattern of the UEs. Therefore, ergonomic factors such as wheel size, operating distance, and operating height are suggested for consideration in CSM analysis. The contribution of different muscles during CSM could be further analyzed using our system with additional EMG measurements. Furthermore, the velocity of wheel rotation might also affect the movement pattern of CSM as the contact duration and hand use might change to adapt to the requirement of continuous motion. In this study, we selected the angular velocity 180°/s which is considered medium to fast for isokinetic exercise [23,24]. These data must be interpreted with caution as the kinetic and kinematic parameters might change when using a slower or faster velocity of wheel rotation, and we may investigate this effect in the future.

In the methodological aspect, several approaches including the preparation work before the test, online monitoring during the test, and verification as well as data processing after the test have been used to enhance the reliability and validity of measurements. These approaches were valid methods to reduce the measurement errors [25,26] and could be recommended for the future quantitative study on driving movement. In pretrial practice, we also noted that some subjects tried to turn the steering wheel with trunk rotation involvement if the subjects sat too far to the wheel. As a previous study showed that trunk involvement might reduce the elbow and shoulder use during the reaching movement [27], we decided to minimize the effect by selecting the proper position of the subject with respect to the steering wheel as well as asking the subject not to leave the seat back during CSM and monitoring them to ensure that they did so. Nevertheless, trunk involvement is allowed and common in real driving situations. The associated effects of trunk involvement on UE use during CSM might need to be studied further in the future.

Obviously, vehicle steering is a complex process that involves the brain, muscles, and limbs in response to demanding driving situations. Among the movements of vehicle steering, CSM might be the more challenging parts as CSM involves a large movement range and intra-limb and inter-limb coordination [28]. We believe CSM could be a good exercise program for pathological patients if the involved muscles under various conditions (considering ergonomic factors and rotating velocity) could be fully understood. Compared to previous systems developed for the purpose of rehabilitation [15,29], our system provides not just the outcome of a steering movement (the position of the steering wheel) but also information on the force exertion and hand contact of the CSM, which can be used for further analysis. In future, we might utilize the information as a feedback signal for movement training of pathological patients suffering from conditions such as traumatic brain injury or stroke. The system might also be used to evaluate the motor ability of pathological subjects who want to return to normal driving as it offers tremendous advantages over traditional driving assessment tools [30–32] in a more objective and reliable manner.

## 5. Conclusions

In this study, we have developed an integrated measurement system to study the continuous steering movement. To our knowledge, no previous study has focused on the movement pattern and force exertion during CSM. We found that CSM is a repeatedly and reciprocally bimanual task with asymmetric contributions from the two hands. This information can be used to develop targeted interventions aimed at improving strength and coordination of the UEs after injuries. A future study investigating the various muscles used during CSM would be very helpful and interesting.

## Figures and Tables

**Table 1.** Participant profiles.

**Subject No.**	**Sex**	**Height (cm)**	**Weight (kg)**	**SJ2SJ (cm)**	**SJ2SC (cm)**	**SJ2FT (cm)**	**SJ2FL (cm)**	**SC2FL (cm)**	**TA (°)**
s1	M	169	50	36.5	53.5	73	78	61.5	75
s2	M	180	70	39	56	78	81	64.5	69
s3	F	170	80	37	50	72	78	62	69
s4	F	156	50	32.5	49	66.5	75.5	64	69
s5	F	157.5	53.5	33	46	65	72	62	74
s6	F	157	50	33.5	49.5	65.5	76	63	74
s7	F	168	58	34	52	74	81	67.5	70
s8	M	170	60	38	54	73.5	76	67.5	70
s9	M	168	59	38	51	73.5	75	67.5	70
s10	M	177	70	40	55	77	79	69	78
s11	F	171	57	35.5	51	72.5	80	67.5	78
s12	F	166	80	37	53	69	80.5	70	69

Mean (SD)		167.5 (7.5)	61.5 (11.0)	36.2 (2.5)	51.7 (2.8)	71.6 (4.3)	77.7 (2.8)	65.5 (3.0)	72.1 (3.5)

SJ2SJ: the distance between the right and left acromia of the shoulder joints; SJ2SC: the distance from the right shoulder to the steering center; SJ2FT: the distance from the right shoulder to the tip of the right middle finger; SJ2FL: the height of the right shoulder with respect to the floor; SC2FL: the height of the steering center with respect to the floor; TA: the angle of tilt of the steering wheel with respect to the horizontal plane.

**Table 2.** Averaged amplitudes of torque during single stance phase (Unit: N·m).

**(n = 12)**		**Mean (SD)**	***P* Value**
CW	Right	8.23 (2.65)	0.001 ^*^
	Left	5.94 (1.51)	
CCW	Right	5.43 (1.42)	<0.001 ^*^
	Left	8.04 (2.42)	

CW: clockwise steering; CCW: counterclockwise steering.

* The symbols denote a significant difference between the torque amplitudes for the left and right hands, as evidenced by the fact that *P* < 0.05.

**Table 3.** Temporal parameters: cycle time, stance time, swing time, and stance ratio during CW and CCW steering.

**(n = 12)**		**Cycle Time (s)**		**Stance Time (s)**		**Swing Time (s)**		**Stance Ratio**	
		**R′t**	**L′t**	**R′t**	**L′t**	**R′t**	**L′t**	**R′t**	**L′t**
CW	Mean (SD)	1.60 (0.25)	1.60 (0.25)	0.87 (0.13)	1.00 (0.17)	0.73 (0.18)	0.61 (0.12)	0.55 (0.06)	0.62 (0.04)
*P* value		0.131		0.027 *		0.031 *		0.019 *	
CCW	Mean (SD)	1.53 (0.25)	1.53 (0.24)	0.95 (0.19)	0.84 (0.13)	0.58 (0.09)	0.69 (0.14)	0.62 (0.05)	0.55 (0.04)
*P* value		0.795		0.006 *		0.007 *		0.007 *	

CW: clockwise steering; CCW: counterclockwise steering.

* The symbols denote a significant difference in parameters between the left and right hands, as evidenced by the fact that *P* < 0.05.
